# Supplementary material for: Pyruvate Dehydrogenase Kinase 1 inhibition mediated oxidative phosphorylation enhancement in cartilage promotes osteoarthritis progression
Source: BMC Musculoskelet Disord. 2023 Jul 20;24:597. doi: 10.1186/s12891-023-06585-6 (PMC10357736; doi:10.1186/s12891-023-06585-6)
Supplement: Supplementary file 1 — Additional file 1. [file 12891_2023_6585_MOESM1_ESM.zip › KEGG.Gsea.1653622432464/KEGG_NON_SMALL_CELL_LUNG_CANCER.html]

Details for gene set KEGG\_NON\_SMALL\_CELL\_LUNG\_CANCER[GSEA]

|  || Dataset | 55457\_collapsed\_to\_symbols.cls.cls #OA\_versus\_normal.cls.cls #OA\_versus\_normal\_repos |
| Phenotype | cls.cls#OA\_versus\_normal\_repos |
| Upregulated in class | normal |
| GeneSet | KEGG\_NON\_SMALL\_CELL\_LUNG\_CANCER |
| Enrichment Score (ES) | -0.2828438 |
| Normalized Enrichment Score (NES) | -1.0997931 |
| Nominal p-value | 0.3018242 |
| FDR q-value | 0.5271349 |
| FWER p-Value | 1.0 |
Table: GSEA Results Summary

  

Fig 1: Enrichment plot: KEGG\_NON\_SMALL\_CELL\_LUNG\_CANCER      
 Profile of the Running ES Score & Positions of GeneSet Members on the Rank Ordered List

  

| SYMBOL | TITLE | RANK IN GENE LIST | RANK METRIC SCORE | RUNNING ES | CORE ENRICHMENT || 1 | PLCG2 | phospholipase C gamma 2 [Source:HGNC Symbol;Acc:HGNC:9066] | 725 | 0.588 | -0.0201 | No |
| 2 | MAPK3 | mitogen-activated protein kinase 3 [Source:HGNC Symbol;Acc:HGNC:6877] | 757 | 0.579 | 0.0133 | No |
| 3 | CDKN2A | cyclin dependent kinase inhibitor 2A [Source:HGNC Symbol;Acc:HGNC:1787] | 799 | 0.570 | 0.0453 | No |
| 4 | ERBB2 | erb-b2 receptor tyrosine kinase 2 [Source:HGNC Symbol;Acc:HGNC:3430] | 963 | 0.526 | 0.0651 | No |
| 5 | AKT1 | AKT serine/threonine kinase 1 [Source:HGNC Symbol;Acc:HGNC:391] | 1192 | 0.478 | 0.0769 | No |
| 6 | CDK4 | cyclin dependent kinase 4 [Source:HGNC Symbol;Acc:HGNC:1773] | 1375 | 0.447 | 0.0904 | No |
| 7 | PIK3CB | "phosphatidylinositol-4,5-bisphosphate 3-kinase catalytic subunit beta [Source:HGNC Symbol;Acc:HGNC:8976]" | 2186 | 0.340 | 0.0484 | No |
| 8 | EGF | epidermal growth factor [Source:HGNC Symbol;Acc:HGNC:3229] | 2399 | 0.315 | 0.0513 | No |
| 9 | FHIT | fragile histidine triad diadenosine triphosphatase [Source:HGNC Symbol;Acc:HGNC:3701] | 2758 | 0.274 | 0.0404 | No |
| 10 | PRKCA | protein kinase C alpha [Source:HGNC Symbol;Acc:HGNC:9393] | 3728 | 0.183 | -0.0237 | No |
| 11 | BAD | BCL2 associated agonist of cell death [Source:HGNC Symbol;Acc:HGNC:936] | 3788 | 0.178 | -0.0173 | No |
| 12 | RARB | retinoic acid receptor beta [Source:HGNC Symbol;Acc:HGNC:9865] | 4166 | 0.141 | -0.0379 | No |
| 13 | E2F1 | E2F transcription factor 1 [Source:HGNC Symbol;Acc:HGNC:3113] | 5137 | 0.061 | -0.1097 | No |
| 14 | TP53 | tumor protein p53 [Source:HGNC Symbol;Acc:HGNC:11998] | 5511 | 0.032 | -0.1368 | No |
| 15 | GRB2 | growth factor receptor bound protein 2 [Source:HGNC Symbol;Acc:HGNC:4566] | 5697 | 0.016 | -0.1502 | No |
| 16 | RB1 | RB transcriptional corepressor 1 [Source:HGNC Symbol;Acc:HGNC:9884] | 6013 | -0.006 | -0.1743 | No |
| 17 | RAF1 | "Raf-1 proto-oncogene, serine/threonine kinase [Source:HGNC Symbol;Acc:HGNC:9829]" | 6187 | -0.019 | -0.1866 | No |
| 18 | PRKCB | protein kinase C beta [Source:HGNC Symbol;Acc:HGNC:9395] | 6453 | -0.039 | -0.2048 | No |
| 19 | MAPK1 | mitogen-activated protein kinase 1 [Source:HGNC Symbol;Acc:HGNC:6871] | 6853 | -0.068 | -0.2317 | No |
| 20 | RXRG | retinoid X receptor gamma [Source:HGNC Symbol;Acc:HGNC:10479] | 6947 | -0.075 | -0.2343 | No |
| 21 | NRAS | "NRAS proto-oncogene, GTPase [Source:HGNC Symbol;Acc:HGNC:7989]" | 7300 | -0.102 | -0.2554 | No |
| 22 | RXRA | retinoid X receptor alpha [Source:HGNC Symbol;Acc:HGNC:10477] | 7381 | -0.109 | -0.2549 | No |
| 23 | PIK3CD | "phosphatidylinositol-4,5-bisphosphate 3-kinase catalytic subunit delta [Source:HGNC Symbol;Acc:HGNC:8977]" | 7405 | -0.111 | -0.2498 | No |
| 24 | CCND1 | cyclin D1 [Source:HGNC Symbol;Acc:HGNC:1582] | 7512 | -0.119 | -0.2507 | No |
| 25 | PIK3CA | "phosphatidylinositol-4,5-bisphosphate 3-kinase catalytic subunit alpha [Source:HGNC Symbol;Acc:HGNC:8975]" | 7733 | -0.136 | -0.2594 | No |
| 26 | PIK3CG | "phosphatidylinositol-4,5-bisphosphate 3-kinase catalytic subunit gamma [Source:HGNC Symbol;Acc:HGNC:8978]" | 7953 | -0.152 | -0.2671 | Yes |
| 27 | HRAS | "HRas proto-oncogene, GTPase [Source:HGNC Symbol;Acc:HGNC:5173]" | 7977 | -0.153 | -0.2594 | Yes |
| 28 | AKT3 | AKT serine/threonine kinase 3 [Source:HGNC Symbol;Acc:HGNC:393] | 8008 | -0.156 | -0.2521 | Yes |
| 29 | SOS2 | SOS Ras/Rho guanine nucleotide exchange factor 2 [Source:HGNC Symbol;Acc:HGNC:11188] | 8058 | -0.160 | -0.2460 | Yes |
| 30 | E2F2 | E2F transcription factor 2 [Source:HGNC Symbol;Acc:HGNC:3114] | 8090 | -0.162 | -0.2384 | Yes |
| 31 | ARAF | "A-Raf proto-oncogene, serine/threonine kinase [Source:HGNC Symbol;Acc:HGNC:646]" | 8092 | -0.163 | -0.2284 | Yes |
| 32 | CASP9 | caspase 9 [Source:HGNC Symbol;Acc:HGNC:1511] | 8142 | -0.166 | -0.2220 | Yes |
| 33 | BRAF | "B-Raf proto-oncogene, serine/threonine kinase [Source:HGNC Symbol;Acc:HGNC:1097]" | 8171 | -0.169 | -0.2137 | Yes |
| 34 | RASSF1 | Ras association domain family member 1 [Source:HGNC Symbol;Acc:HGNC:9882] | 8229 | -0.173 | -0.2075 | Yes |
| 35 | CDK6 | cyclin dependent kinase 6 [Source:HGNC Symbol;Acc:HGNC:1777] | 8393 | -0.185 | -0.2087 | Yes |
| 36 | PRKCG | protein kinase C gamma [Source:HGNC Symbol;Acc:HGNC:9402] | 8582 | -0.200 | -0.2110 | Yes |
| 37 | TGFA | transforming growth factor alpha [Source:HGNC Symbol;Acc:HGNC:11765] | 8748 | -0.212 | -0.2107 | Yes |
| 38 | PIK3R3 | phosphoinositide-3-kinase regulatory subunit 3 [Source:HGNC Symbol;Acc:HGNC:8981] | 8801 | -0.217 | -0.2014 | Yes |
| 39 | PIK3R5 | phosphoinositide-3-kinase regulatory subunit 5 [Source:HGNC Symbol;Acc:HGNC:30035] | 8804 | -0.217 | -0.1881 | Yes |
| 40 | MAP2K2 | mitogen-activated protein kinase kinase 2 [Source:HGNC Symbol;Acc:HGNC:6842] | 9932 | -0.315 | -0.2564 | Yes |
| 41 | MAP2K1 | mitogen-activated protein kinase kinase 1 [Source:HGNC Symbol;Acc:HGNC:6840] | 10197 | -0.340 | -0.2559 | Yes |
| 42 | KRAS | "KRAS proto-oncogene, GTPase [Source:HGNC Symbol;Acc:HGNC:6407]" | 10544 | -0.377 | -0.2595 | Yes |
| 43 | PLCG1 | phospholipase C gamma 1 [Source:HGNC Symbol;Acc:HGNC:9065] | 10722 | -0.395 | -0.2489 | Yes |
| 44 | PIK3R2 | phosphoinositide-3-kinase regulatory subunit 2 [Source:HGNC Symbol;Acc:HGNC:8980] | 10956 | -0.425 | -0.2407 | Yes |
| 45 | STK4 | serine/threonine kinase 4 [Source:HGNC Symbol;Acc:HGNC:11408] | 11074 | -0.441 | -0.2226 | Yes |
| 46 | PIK3R1 | phosphoinositide-3-kinase regulatory subunit 1 [Source:HGNC Symbol;Acc:HGNC:8979] | 11099 | -0.443 | -0.1970 | Yes |
| 47 | FOXO3 | forkhead box O3 [Source:HGNC Symbol;Acc:HGNC:3821] | 11305 | -0.472 | -0.1838 | Yes |
| 48 | PDPK1 | 3-phosphoinositide dependent protein kinase 1 [Source:HGNC Symbol;Acc:HGNC:8816] | 11839 | -0.557 | -0.1909 | Yes |
| 49 | RXRB | retinoid X receptor beta [Source:HGNC Symbol;Acc:HGNC:10478] | 11871 | -0.565 | -0.1584 | Yes |
| 50 | SOS1 | SOS Ras/Rac guanine nucleotide exchange factor 1 [Source:HGNC Symbol;Acc:HGNC:11187] | 12041 | -0.603 | -0.1342 | Yes |
| 51 | EGFR | epidermal growth factor receptor [Source:HGNC Symbol;Acc:HGNC:3236] | 12109 | -0.622 | -0.1010 | Yes |
| 52 | E2F3 | E2F transcription factor 3 [Source:HGNC Symbol;Acc:HGNC:3115] | 12886 | -1.224 | -0.0857 | Yes |
| 53 | AKT2 | AKT serine/threonine kinase 2 [Source:HGNC Symbol;Acc:HGNC:392] | 12896 | -1.404 | 0.0003 | Yes |
Table: GSEA details [plain text format]

  

Fig 2: KEGG\_NON\_SMALL\_CELL\_LUNG\_CANCER      
 Blue-Pink O' Gram in the Space of the Analyzed GeneSet

  

Fig 3: KEGG\_NON\_SMALL\_CELL\_LUNG\_CANCER: Random ES distribution      
 Gene set null distribution of ES for **KEGG\_NON\_SMALL\_CELL\_LUNG\_CANCER**
